# Medical waste management improvement in community health centers: an interventional study in Iran

**DOI:** 10.1017/S1463423618000622

**Published:** 2018-08-20

**Authors:** Jafar Sadegh Tabrizi, Mohammad Saadati, Mahdieh Heydari, Ramin Rezapour, Roghaie Zamanpour

**Affiliations:** 1 Department of Health Service Management, Tabriz Health Service Management Research Centre, School of Health Management and Medical Informatics, Tabriz University of Medical Sciences, Tabriz, Iran; 2 Road Traffic Injury Research Center, Tabriz University of Medical Sciences, Tabriz, Iran; 3 Iranian Center of Excellence in Health Management, School of Health Management and Medical Informatics, Tabriz University of Medical Sciences, Tabriz, Iran; 4 Environmental Health Engineer, Health Affair, Tabriz University of Medical Sciences, Tabriz, Iran

**Keywords:** clinical audit, community health center, medical waste, process improvement

## Abstract

**Aim:**

To improve the medical waste management (MWM) standards in Tabriz community health centers (CHCs) through clinical audit process.

**Background:**

Management of medical waste is not only a legally necessity but also a social responsibility in health systems. Owing to the potential risks for human health and environmental impacts, MWM is a global concern.

**Methods:**

This was an interventional research designed using clinical audit cycle that was implemented in Tabriz CHCs in 2016. MWM was assessed through observation, as well as reviewing relevant documents and interviews with waste workers in CHCs and completion of a researcher-made checklist. Intervention plans were developed and implemented based on the assessment results. To analyze the data, Excel 2016 software was used and information was reported as descriptive statistics through comparison of standards adherence before and after the interventions.

**Results:**

Generally, 30% improvements in MWM standards adherence were experienced (45.8–75.1%) in the CHCs, after the interventions. The greatest improvement was observed in the dimensions of management and education, and separation and collection of medical waste, up to 30 and 28.5%, respectively.

**Conclusions:**

As the results demonstrated, standards of MWM processes were improved in Tabriz CHCs, due to the intervention. Moreover, it was experienced that using systematic method, stakeholders’ participation and evidence-based planning would lead to process improvement. MWM was an ignored issue in primary care that must be more in attention.

## Background

Health services delivery can generate various kinds of hazardous wastes called ‘medical waste’ (Ali *et al*., 2017). Medical waste was defined as solid or semi-solid waste produced over diagnosis and/or treatment process (Rau *et al*., 2000). Medical wastes include infectious waste containing 10–25% of the waste (Graikos *et al*., 2010) and non-infectious waste which accounts for 75–95% of the entire waste (Askarian *et al*., 2010). Infectious waste and sharps pose the highest level of risk threatening the health of the staff of medical centers and people visiting as they put them at risk of diseases such as AIDS, hepatitis B and C (Mandal and Dutta, 2009). It is surprising that health care providers whose intention is to offer people medical services and protect them from diseases have now become a source of infection (Hanumantha Rao, 2008; Sadeghi-Bazargani *et al*., 2015; Saadati *et al*., 2017). Therefore, medical waste is one of the special dimensions of municipal solid waste that are of great importance due to their hazardous and infectious nature (Mandal and Dutta, 2009). It is not only legally required to manage medical waste effectively and in absolute security but it is a social responsibility as well (Bencko *et al*., 2003) as this process deals with a wide range of employees, patients and other people’s health and safety (Patil and Pokhrel, 2005). In order to protect the environment and due to safety issues, it is of great importance to regard how to collect, separate, transport, store, dispose and treat medical waste (Rao *et al*., 2004). Thus, poor management of medical waste will result in undesirable environmental impacts (Jang *et al*., 2006; Chartier, 2014; Windfeld and Brooks, 2015).

Many countries still do not have the proper rules on medical waste management (MWM) and if they have, they do not actually put them into practice (Babanyara *et al*., 2013). According to studies conducted on MWM in some Iranian cities, including Tehran, Mashhad, Kashan and Rasht, despite the existence of laws in this regard, failure to perform and comply with them and poor inspection of the Ministry of Health have led to an inefficient management process (Koushiar *et al*., 2006; Masoumbeigi *et al*., 2007; Arab *et al*., 2008).

On the contrary, the high cost differences between the disposal of infectious waste and non-infectious waste is another important economic factor that must be considered in health care facilities (Rushbrook, 2004). Thus, highly advanced and costly refinement and filtration methods are required to treat infectious and chemical waste of health centers. Therefore, preventive and controlling measures to reduce and minimize the production of hazardous waste in health centers are one of the main strategies of the World Health Organization for developing countries (Prüss-Üstün *et al*., 1999). Through an appropriate management system, the medical waste production rate could be reduced up to 15%, which in turn could drop the environmental health problems caused by these waste (Alagöz and Kocasoy, 2008). Another issue is that waste management is usually delegated to workers who do their tasks mainly without proper instructions or adequate support (Diaz *et al*., 2005). Since infectious waste contain large amounts of contagious pathogens, it is much likely that predisposed people get infected when they touch these wastes (Bdour *et al*., 2007). That is why MWM is one of the main factors affecting development infrastructure that needs the special attention of health policymakers and managers (Mandal and Dutta, 2009). Lack of equipment, inadequate physical spaces for storage, poor waste separation, and unsafe sterilization and disposal were among the most important issues of MWM in health centers reported in the literature (Alagöz and Kocasoy, 2008; Shinee *et al*., 2008; Ruoyan *et al*., 2010). Moreover, previous studies in Iran had revealed that the MWM in primary health care centers is faced with several problems such as low knowledge of the staff, inappropriate separation and treatment and then needs to be improved (Mesdaghinia *et al*., 2009).

Clinical audit is one of the most effective quality improvement methods, which is focused on standards. This is done through a systematic assessment of the *status quo* and adapting it to explicit standards and then employing interventions that result in changes (Excellence, 2002). Tabriz is the largest city in the northwest of Iran that offers a wide range of health care services to a large population. Although the status of MWM in Tabriz health centers was described in previous studies, to the best of authors’ knowledge, no interventional study has been performed in this regard (Tabrizi *et al*., 2018). So, this study aimed to improve the MWM standards in Tabriz community health centers (CHCs) through clinical audit.

## Methods

**Figure 1:**
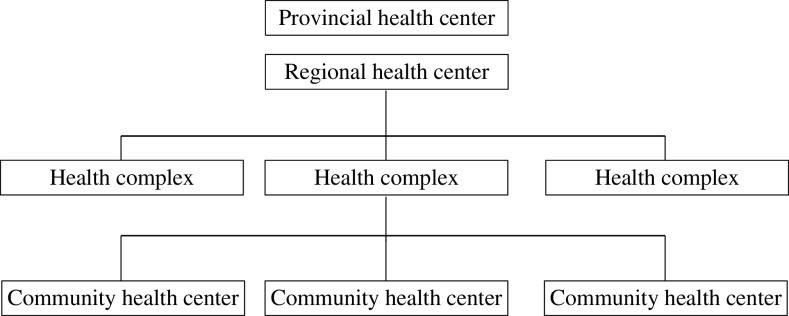
Organization chart of primary health care system

The present study was an interventional research conducted using the clinical audit cycle designed and implemented in Tabriz CHCs in 2016–2017. Descriptive part of the study had already been developed and published in a research paper, which included the first four phases of clinical audit cycle (subject selection, standard setting, reviewing the existing status and comparing it with standards) performed in 57 urban CHCs (Tabrizi *et al*., 2018). After Iran’s Health System Reform Plan implemented in 2016, the entire structure of the primary health care system was changed in Tabriz. As a result of such reform, 20 health complexes were established as staffing and supporting units, each of which comprised three to five CHCs (87 CHCs overall) providing primary care services. Among all health complexes in Tabriz, 11 were publicly owned and the rest are private (Figure 1).

So, the two stages of clinical audit (evaluation of *status quo* and comparing it with standards) were repeated to determine new *status quo* of CHCs. The used model of clinical audit process has six stages of which stages 3 and 4 were repeated after the intervention. The stages of clinical audit were carried out as follows:

### Audit subject

MWM process in Tabriz CHCs.

### Used standards

These include MWM methods and process standards approved by Commission on Infrastructure Affairs of the Islamic Consultative Assembly and Environmental Protection Organization of Iran (Number: 1901/56061) (Islamic Consultative Assembly, 2008). The standards were converted to a checklist by the research team and used to collect data after its content validity was confirmed by relevant experts (*n*=6). The checklist consisted of four dimensions, including management and training, separation and collection, transportation and temporary storage, and sterilization and disposal.

### Evaluation of *status quo*

To examine the status of MWM process in CHCs, the assessment team was formed with the participation of environmental health officers of the health complexes (*n*=20). First, the officers were provided with training sessions on standards and how to complete the checklist. Then, the assessment program was developed upon which the environmental health officers of the health complexes evaluated the *status quo* of MWM in CHCs under their own surveillance in August 2016. They have reported the assessment results to the Tabriz regional health center officers. The assessment was carried out by observing the ongoing processes along with reviewing the relevant documentations, interviews with process owners (waste workers and other related staff) in CHCs and then completing the checklist. Checklist questions were scored by assigning number one to standards adherence and zero to non-adherence. Next, the total score of all four dimensions was calculated and divided by the number of questions of each dimension to obtain the standard adherence both in general and separately for each dimension. Regarding that the dimension ‘sterilization and disposal’ only took place in Tabriz regional health center after collection of the waste from all CHCs in the city, data on this stage were only gathered and analyzed at this level. To verify the validity of the assessments, an external expert in the field of environmental health along with an expert from the Tabriz regional health center, randomly re-evaluated some CHCs.

### Comparing baseline status and standards’ set

After all CHCs in Tabriz were assessed, data were entered into Excel 2016 software and then adherence of existing status with standards was examined and represented as charts using descriptive statistics. Afterwards, reports were extracted and developed in three levels of Tabriz city, health complexes and CHCs in order to understand the current situation and to adopt appropriate strategies to improve MWM standards. To facilitate the evaluation and comparisons, a database analyzing and presenting comparative graphs at CHCs and health complexes levels was designed using the Excel 2016 software.

### Interventions’ design and implementation

In this step, a comprehensive report on MWM standards compliance and solutions was prepared by the officers of each health complexes and presented at a meeting in the presence of provincial and regional environmental health authorities. In four groups then, the environmental health officers discussed the suggested solutions and proposed new ones to improve the MWM at two levels of health complexes and CHCs. Then the provided solutions were prioritized using a prioritization matrix. The proposed solutions of each group were revealed to other groups. Finally, after integrating and modifying all solutions, a single list of 13 highly prioritized interventions was prepared. Subsequently, interventions’ action plan was developed with participation of environmental health officers and then announced to all CHCs by Tabriz regional health center. The action plan implementation was carried out involving all staff in CHCs.

### Re-audit

The improvement level of MWM standards in health complexes and CHCs was monitored on a monthly basis using assessment database by environmental health officers. Re-audit plan was scheduled in the action plan in two different periods, two and four months after the interventions’ execution. Results of each assessment were compared with pre-intervention results using trend charts and radar charts. Moreover, to study the sustainability of the changes, an assessment was planned for one year after the intervention.

## Results

The results of assessment in August 2016 indicated that 55% of the standards were not adhered in CHCs. According to the results, weaknesses of CHCs were identified and adjustment interventions were designed for each of four dimensions of MWM through workgroups of MWM officers of the CHCs (Table 1). Action plan was developed and implemented by participation of all stakeholders of MWM in the CHCs.

**Table 1:** Issues and adjustment interventions classified by dimensions of medical waste management (MWM)

*N*	Dimensions	Issues	Interventions
1	Management and training	Lack of professional trainings on MWM No educational packages Lack of comprehensive MWM program	Holding two professional workshops for related staff Publishing educational booklets and posters based on standards Development of MWM program in CHCs
2	Separation and collection	Lack of waste separation equipment (bag and bins) Lack of labels indicating the type of waste and its characteristics on bins Incorrect waste separation by the health staff	Providing yellow bins and plastic bags (for infectious waste) and blue bins and black plastic bags (for non-infectious waste) as many as needed Designing and sticking labels on yellow and blue bins indicating infectious waste and treated waste, respectively Regular monitoring of waste separation using the labels
3	Transporting and temporary storage	Lack of temporary storage spaces Lack of regular schedule for the waste transferring Non-assignment of a special vehicle for the waste transferring	Equipping CHCs with medical waste trolleys (considering the low volume of waste and lack of standard temporary storage facilities in the CHCs) Monthly scheduling to regularly collect and transfer the medical waste from CHCs Purchasing special vehicles with standard characteristics for transportation of medical waste
4	Sterilization and disposal	Personal protection equipment’s shortage Deficiency of sanitary facilities at disposal location Have no cold storage system in CHCs Air pollution due to waste treatment Low knowledge of the autoclave operator Failure to monitor and evaluate the function of the autoclave Waste disposal stops in device failure times	Providing personal protection equipment’s for disposal staff Improving sanitary facilities in place such as floor washing equipment, etc. Construction and equipping of cold storage space in Tabriz regional health center Installing filtration system to decrease air pollutions caused by treatment process Scheduling daily sterilization of medical waste by a full-time operator Contracting with a local hospital as surrogate for waste disposal in device failure times

CHCs=community health centers.

**Figure 2:**
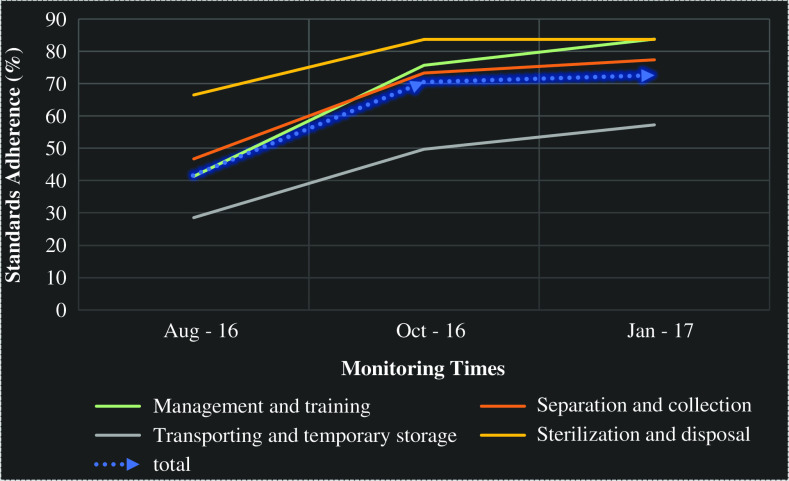
Medical waste management standards adherence improvement trend in Tabriz community health centers – dimension and overall

According to the action plan, the first and second assessments must be conducted at two and four months, respectively, after interventions began. The results of baseline assessment performed in August 2016 indicated that 40% of CHCs have <25% standards compliance in the management and training dimension (Table 2). Re-assessment results, in January 2017, showed a 42.5% difference in standards adherence (Figure 2).

**Table 2:** Comparison of standards adherence before and after intervention in community health centers

	Management and training (%)			Separation and collection (%)			Transporting and temporary storage (%)			Total (%)		
	⩽25	25–50	⩾50	⩽25	25–50	⩾50	⩽25	25–50	⩾50	⩽25	25–50	⩾50
Before (*N*=57)	40	25	35	10	55	35	60	25	15	30	40	30
After (*N*=87)	0	0	100	0	0	100	0	0	100	0	0	100

Process improvement monitoring results, in October 2016 and January 2017, revealed that adherence with waste separation and collection standards by CHCs was improved by 30.7% and all of the CHCs standards compliance grew more than 50% (Figure 2).

Before the intervention, transportation and temporary storage standards compliance rate in 60% of the CHCs was <25%, which was improved and none of the CHCs were in <50 status, after intervention (Table 2).

Generally, before the interventions, 30% of CHCs were under 25%, and 40% of them were between 25 and 50% in terms of standards adherence in the three dimensions. The evaluation performed in January 2017 revealed that standards adherence by CHCs was improved on average by 38.7% and all CHCs were classified in upper 50% category (Table 2).

Regarding the fact that sterilization and disposal process was conducted in Tabriz regional health center medical waste treatment site, this part was only investigated at this facility. The process assessment before intervention showed 66% standards adherence which rose up to 83.8% after the interventions was executed. This means that the interventions have made a 17.2% improvement in sterilization and disposal process (Figure 2).

**Figure 3:**
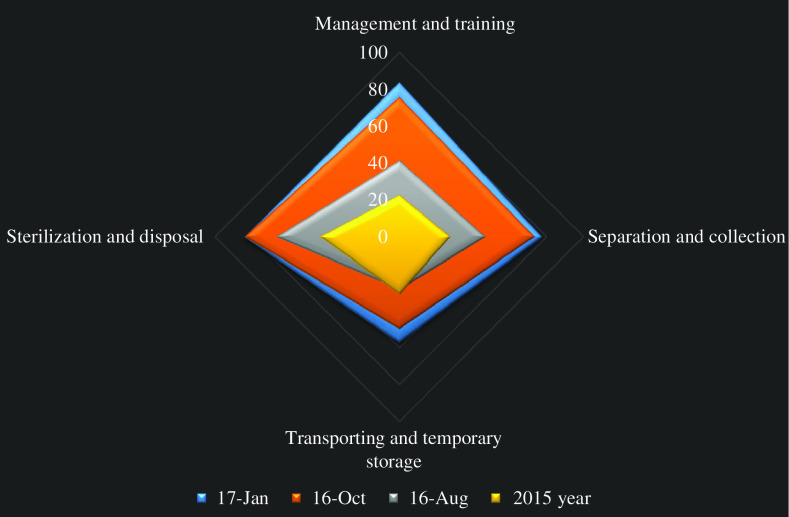
Medical waste management improvement in Tabriz community health centers over the four dimensions of the assessment

Comparing the MWM standards adherence rate in CHCs before and after intervention illustrated that they experienced the highest level of improvement in the management and training dimension as well as separation and collection of medical waste. Figure 3 represents the adherence rate with MWM standards in CHCs over the four stages of assessments (two stages before intervention and two afterwards).

## Discussion

This study demonstrated that systematic and scientific supports in primary health care system would lead to quality improvement in MWM processes. As was experienced in this study, 30% improvement in MWM standards adherence (45.8–75.1%) was evidenced in Tabriz CHCs. This achievement was based on four major key factors: staff participation, using systematic quality improvement method, evidence-based interventions and leadership commitment and support. Similarly, waste management improvement programs implemented in a Brazilian primary health care center were successful (Moreira and Günther, 2013). Using systematic and significantly supported quality improvement methods in primary health care would lead to more responsiveness of the primary health systems in countries.

Interventions implemented in this study were not only supported by the assessment data but also by previous literature in Iran. Staff knowledge deficiency about waste management process was raised as one of the most important problems in Iran’s primary health care centers (Mesdaghinia *et al*., 2009). The baseline rate of standards adherence in management and training dimension was 22.8% (in January 2015) and 41.4% (in August 2016) in Tabriz CHCs. This was consistent with the result of Amouei *et al*. (2015), which was showed a low level of knowledge in hospital personnel. Literature had demonstrated that where personnel knowledge is poor, it is not expected to perform well (Moreira and Günther, 2013; Hakim *et al*., 2014). However, promoting staff knowledge and skills is not the only condition to improve their performance. Closely monitoring the provided training courses and their performance level is another matter that should be addressed by managers exactly as emphasized in previous studies (Yadavannavar *et al*., 2010). Such results in our study was due to lack of proper trainings for staff, which was improved up to 83.9% adherence after interventions through providing appropriate training pamphlets and packages, holding training courses for the staff involved with the process, especially waste workers. Staff participation in developing the educational packages and holding educational classes, in this study, was experienced to be effective in successful implementation of the interventions.

Medical waste separation and collection is the most important step in waste management. Previous studies in Iran have documented that only 25% of primary health care centers separate their hazardous waste correctly (Mesdaghinia *et al*., 2009). Our study results clarified that, before intervention, about 35% of CHCs comply with more than 50% of the standards in this dimension. Literature had reported that, in Ardebil and Tehran primary health care centers, 65.4 and 96%, respectively, had complied with standards of waste separation and collection which was higher than our results (Sadeghi *et al*., 2012; Sepehrnia *et al*., 2016). Medical waste separation must be done in the point of generation in health centers (Jang *et al*., 2006). Hence, providing proper facilities and equipment for standard separation is a necessity in CHCs. So adjustments such as providing standard color-coded bags and containers and bin labeling were developed and implemented in CHCs. Improvement in separation and collection standards adherence in health centers leads to better material recycling (Jang *et al*., 2006). On the contrary, correct separation of the waste in generation points reduces the marginal cost of waste management in health system.

According to the evidences, lack of physical site for temporary storage, failure to comply with the standard duration of infectious waste temporary storage in CHCs and low education level of the waste workers who were responsible for collection and transportation of medical waste were among the most important reasons causing failure in temporary storage of medical waste in Iran (Mesdaghinia *et al*., 2009; Sadeghi *et al*., 2012). These issues were raised in our study too and adjustment interventions were developed to resolve them. Accordingly, about 28.7% improvement in temporary storage standards in CHCs was experienced after intervention. However, the transportation and temporary storage dimension had the lowest standard adherence. Considering that medical waste must be separated as infectious and non-infectious waste, their temporary storage also must be done separately. This is dependent on health centers ability to provide adequate physical space (Mesdaghinia *et al*., 2009). Unsafe sterilization and disposal of medical waste in primary health centers was reported by several studies (Mesdaghinia *et al*., 2009; Ruoyan *et al*., 2010; Moreira and Günther, 2013).

Our intervention plan led to 17.2% standards promotion in medical waste disposal process in Tabriz regional health center. Medical waste disposal inadequacy was mostly due to inappropriate autoclave process and equipment. As an important issue in medical waste disposal, autoclave performance control using Bowie-Dick test was suggested by literature to ensure the sterilization process which was ignored in Tabriz regional health center (Crossley, 2014). The Bowie-Dick test is widely used and recognized as a valuable means of monitoring the performance of vacuum-assisted steam sterilizers(Gupta and Shukshith, 2016). The Bowie-Dick test consisted of small disposal thermo-chromatic (temperature-sensitive) paper packs squeezed between porous substrates and reticulated foam. Running the test is in a way that a pack is placed by the autoclave operator in an empty chamber on the lowest shelf above the drain. A Bowie-Dick test pack that shows a color change to dark black color pattern indicates a successful vacuum and full steam penetration. It is conducted every day, before the first process load, because it is a sensitive and rapid means of detecting air leaks, inadequate air removal, inadequate steam penetration and non-condensable gases. Insufficient air removal in a dynamic air-removal sterilizer, particularly a pre-vacuum cycle, can defeat sterilization and result in non-sterile supplies if undetected (Dion and Parker, 2013). Generally, CHCs’ staff had a role in all over the waste management process. This highlights the importance of their knowledge, attitude and practice improvement to have a safe and high-quality waste management.

## Conclusion

MWM standards improved about 30% in Tabriz CHCs. Using systematic and scientific quality improvement method – clinical audit, staff participation, leadership commitment and evidence-based decision-making would lead to primary health care processes quality improvement. This study has provided a scientific approach to quality improvements in MWM in primary health care centers, which has the capability to expand in other cities of Iran or other countries.

## Strengths

This study was conducted using a systematic and scientific method and supported by the city health managers. All stakeholders in the MWM process participated in the development and implementation of interventions. It also covered all CHCs in Tabriz city.

## Limitations

We do not have control group in the study. Moreover, due to the high cost of some interventions such as temporary storage space construction, they were not implemented.
